# Insect-Specific Flavivirus Replication in Mammalian Cells Is Inhibited by Physiological Temperature and the Zinc-Finger Antiviral Protein

**DOI:** 10.3390/v13040573

**Published:** 2021-03-29

**Authors:** Agathe M.G. Colmant, Jody Hobson-Peters, Teun A.P. Slijkerman, Jessica J. Harrison, Gorben P. Pijlman, Monique M. van Oers, Peter Simmonds, Roy A. Hall, Jelke J. Fros

**Affiliations:** 1 Australian Infectious Diseases Research Centre, School of Chemistry and Molecular Biosciences, The University of Queensland, St Lucia 4072, Australia; agathe.colmant@uq.net.au (A.M.G.C.); j.peters2@uq.edu.au (J.H.-P.); jessica.harrison@uqconnect.edu.au (J.J.H.); 2 Laboratory of Virology, Wageningen University and Research, 6708 PB Wageningen, The Netherlands; teunslijkerman@gmail.com (T.A.P.S.); gorben.pijlman@wur.nl (G.P.P.); monique.vanoers@wur.nl (M.M.v.O.); 3 Nuffield Department of Medicine, Peter Medawar Building for Pathogen Research, University of Oxford, Oxford OX1 3SY, UK; peter.simmonds@ndm.ox.ac.uk

**Keywords:** insect-specific flavivirus, CpG, Dinucleotides, innate immunity, zinc-finger antiviral protein

## Abstract

The genus *Flavivirus* contains pathogenic vertebrate-infecting flaviviruses (VIFs) and insect-specific flaviviruses (ISF). ISF transmission to vertebrates is inhibited at multiple stages of the cellular infection cycle, via yet to be elucidated specific antiviral responses. The zinc-finger antiviral protein (ZAP) in vertebrate cells can bind CpG dinucleotides in viral RNA, limiting virus replication. Interestingly, the genomes of ISFs contain more CpG dinucleotides compared to VIFs. In this study, we investigated whether ZAP prevents two recently discovered lineage II ISFs, Binjari (BinJV) and Hidden Valley viruses (HVV) from replicating in vertebrate cells. BinJV protein and dsRNA replication intermediates were readily observed in human ZAP knockout cells when cultured at 34 °C. In ZAP-expressing cells, inhibition of the interferon response via interferon response factors 3/7 did not improve BinJV protein expression, whereas treatment with kinase inhibitor C16, known to reduce ZAP’s antiviral function, did. Importantly, at 34 °C, both BinJV and HVV successfully completed the infection cycle in human ZAP knockout cells evident from infectious progeny virus in the cell culture supernatant. Therefore, we identify vertebrate ZAP as an important barrier that protects vertebrate cells from ISF infection. This provides new insights into flavivirus evolution and the mechanisms associated with host switching.

## 1. Introduction

The genus *Flavivirus* (family *Flaviviridae*) contains many vertebrate-infecting flaviviruses (VIF), including severe human pathogens such as the dengue viruses, Zika virus, yellow fever virus, Japanese encephalitis virus and West Nile virus (WNV). Most VIFs are transmitted between vertebrate hosts by arthropod vectors (e.g., mosquitoes or ticks); however, for a subset of VIFs termed the no known vector flaviviruses (NKVF), replication in arthropod vectors has not been observed. In addition to the VIFs, the *Flavivirus* genus includes many insect-specific flaviviruses (ISF). These viruses have been isolated from mosquitoes and empirical evidence suggests these exclusively replicate in cells of invertebrate origin (reviewed in [1]). Phylogenetic analysis clusters the ISFs in two separate clades. Lineage I ISFs (or classical ISFs) are a clearly distinct group that is relatively divergent from VIFs, while lineage II ISFs (also called dual-host affiliated ISFs) cluster with VIFs [1,2,3,4]. Novel ISFs are still being discovered in mosquitoes and multiple studies have now provided convincing experimental evidence that despite their close relation to VIFs, lineage II ISFs replicate exclusively in invertebrate cells [3,5,6,7,8,9]. Replication of lineage I ISFs in vertebrate cells is inhibited at multiple stages of the cellular infection cycle including viral entry, initiation of viral RNA replication and virus assembly and release from the cell [10,11]. Extensive studies with the recently discovered lineage II ISF Binjari virus (BinJV) revealed that BinJV structural proteins allowed the relatively inefficient entry of virus particles into vertebrate cells. However, BinJV was unable to subsequently initiate viral RNA replication and produce progeny infectious virus. Only when temperatures were reduced from 37 to 34 °C, mouse embryonic fibroblasts (MEF) with the interferon receptor (IFNAR) or endonuclease RNase L knocked out and RIG-I deficient BSR cells displayed low levels of BinJV protein expression [3,12]. Together, this indicates that ISF replication in vertebrate cells is restricted at multiple stages of infection and suggests that vertebrate antiviral pathways may additionally limit lineage II ISF RNA replication inside the vertebrate cell.

The flavivirus genome is a single-strand positive-sense RNA molecule of approximately 11 kilobases in length. The RNA is capped at the 5′-end and is translated into a single polyprotein. This polyprotein is proteolytically processed by cellular and viral proteases which yields three structural proteins; capsid (C), membrane precursor (prM) and envelope (E) and seven nonstructural proteins (NS) [13]. Although the general genetic make-up is similar for all members of the *Flavivirus* genus, ISFs do contain molecular features that are distinct from VIFs. One of the most notable differences between VIFs and ISFs lies in their genome composition, specifically in their CpG dinucleotide usage. Most viruses with RNA genomes have evolved to mirror the dinucleotide usage of their host’s mRNA [14,15]. In vertebrate animals, the cytosine of a CpG dinucleotide is prone to methylation, which can cause it to deaminate and mutate into a thymine [16,17]. In contrast, the genome of most invertebrates contains largely unbiased frequencies of CpG dinucleotides, reflecting greatly reduced DNA methylation activity [18]. CpGs in viral RNA are not subjected to the same methylation and mutation pressure that acts on vertebrate host DNA. Vertebrate cells express the zinc-finger antiviral protein (ZAP), which specifically binds CpG dinucleotides in (viral) single-stranded RNA [19,20,21,22]. Detection by ZAP can effectively attenuate virus replication and a growing number of viruses have been exposed to the ZAP-mediated antiviral responses by experimentally increasing CpG dinucleotide frequencies [19,23,24,25,26]. The genome composition of VIFs resembles that of the vertebrate host with suppressed CpG dinucleotide frequencies, whereas the genomes of ISFs display less suppression of CpG dinucleotides and more closely resembles the CpG usage of their invertebrate host [1,27]. Here, we investigate whether the vertebrate ZAP protein acts as a barrier which senses CpG-high, non-self RNA to protect vertebrate cells from lineage II ISF infection.

## 2. Materials and Methods

### 2.1. Cells and Viruses

Human lung carcinoma A549 cells, A549 ZAP knockout cells and A549 NPro cells [20] were cultured in Dulbecco modified Eagle medium (DMEM) with 10% fetal bovine serum (FBS), penicillin (100 U/mL) and streptomycin (100 µg/mL) and maintained as a monolayer in T25 cell culture flasks at 37 °C with 5% CO_2_. *Aedes albopictus* C6/36 mosquito cells were cultured in Leibovitz L-15 medium (Gibco, Thermo Fischer Scientific, Breda, The Netherlands) supplemented with 10% fetal bovine serum (FBS) (Gibco), 2% tryptose phosphate broth (Gibco), 1% nonessential amino acids (Gibco) and streptomycin (100 g/mL; Sigma-Aldrich, Zwijndrecht, The Netherlands) or Roswell Park Memorial Institute medium (RPMI) with 5% FBS, penicillin (50 U/mL), streptomycin (50 µg/mL) and 2 mmol/L L-glutamine and maintained as a monolayer in cell culture flasks at 28 °C.

Passage 5 USUV stock, the Netherland 2016 black bird isolate (lineage Africa 3, GenBank accession no. MH891847.1) was grown and titrated on Vero E6 cells. WNV Kunjin (strain MRM61C GenBank accession no. KX394398.1 unknown passage number), lineage II ISFs BinJV (GenBank accession no. MG587038.1, passage 2) and Hidden Valley virus (HVV) (GenBank accession no. MN954647.1, passage 3) stocks were propagated on C6/36 cells. Chimeric viruses containing and either the structural genes (prM-E) from BinJV in a WNV Kunjin backbone or WNV structural genes in a BinJV backbone were constructed previously by circular polymerase extension reaction (CPER) [3,5]. Passage 1 virus stocks were grown on C6/36 cells.

### 2.2. In Silico Analysis

Flavivirus genome sequences were selected per flavivirus species and classified as tick-borne, mosquito-borne or NKVF VIF or lineage I/II ISFs. Accession numbers are available in Table 1. Dinucleotide frequencies were calculated with a composition scan using SSE software (v.1.4) [28].

### 2.3. Immunofluorescence

Cells were seeded in an 8-well Lab-Tek™ II Chambered Coverglass system (Nunc, Sigma-Aldrich, Zwijndrecht, The Netherlands) and left to attach overnight. Cells were infected with either USUV or BinJV at MOI 10 and where indicated cells were infected in the presence of 2 μM C16 (Sigma-Aldrich, Zwijndrecht, The Netherlands) in DMSO or solely DMSO. Four days post infection, the cells were fixed with 4% paraformaldehyde solution in PBS for 10 min at room temperature (RT) and subsequently permeabilized with 0.1% SDS in PBS for 10 min at RT. The monolayers were incubated with monoclonal antibodies diluted in a solution of 0.5% skimmed milk powder (Nutricia, Zoetermeer, The Netherlands) in PBS for 1 h at 37 °C. Flavivirus envelope protein was detected with previously described monoclonal antibody BJ-6E6 (hybridoma supernatant diluted 1:3) and dsRNA replication intermediates with either 3G1 (hybridoma supernatant diluted 1:10) or J2 (Scicons, Budapest, Hungary) (diluted 1:100) [3]. Samples were washed three times with PBS and stained with secondary goat anti-mouse Alexa 488 (A-11001; Abcam, Cambridge, United Kingdom) diluted 1:2000 in a solution of 0.5% skimmed milk powder in PBS for 1 h at 37 °C, and the nuclei were stained with a solution of 10 ng/mL Hoechst 33,258 in PBS for 2 min at RT. The cells were visualized by fluorescence microscopy using an Axio Observer Z1m inverted microscope (Zeiss, Jena, Germany) in combination with an X-Cite 120 series lamp.

### 2.4. Viral Replication Assays

For the viral replication kinetics analysis with USUV and BinJV, three biological repeat experiments were performed in which cells were infected in a 24-wells format with MOI 1 at 28 or 34 °C for mosquito and human cells, respectively. After two hours, the inoculum was removed and cells were washed once with PBS before adding fresh cell culture medium. The 50% tissue culture infectious doses (TCID_50_) of cell culture supernatant samples were determined using end-point dilution assays on C6/36 cells. C6/36 monolayers were detached from their plates and diluted tenfold in Leibovitz L-15 cell culture medium. In 96-well plate format, 10-fold serial dilutions of samples were mixed with 90 μL of the C6/36 cell suspension. Five days post-infection, plates were scored based on cytopathic effects (CPE) and by an enzyme-linked immunosorbent assay (ELISA) using BJ-6E6 (BinJV and USUV). For ELISA, cells were fixed by 4% paraformaldehyde in PBS on ice for 10 min and permeabilised by 0.1% SDS in PBS at RT for 10 min. Samples were subsequently blocked with 0.5% skimmed milk powder in PBS for 1 h at RT. Primary antibody 6E6 was diluted in blocking buffer incubated for 1 h at 37 °C. Samples were washed four times with PBS-Tween (0.05%) and incubated with secondary antibody conjugate (Dako goat anti-mouse HRP) diluted 1:2000 in blocking buffer for 1 h at 37 °C. Plates were subsequently washed 6 times with PBS-Tween. ABTS substrate was added and incubated at RT for 1 h in the dark before the TCID_50_ was determined.

For the virus infections with BinJV, HVV, WNV and the chimeric viruses, cells were inoculated with virus in triplicates in 24-well plates at a MOI 0.1, 1 or 10 with 250 μL of inoculum, rocked at RT for 30 min then incubated at 28, 34 or 37 °C for one hour. The inoculum was removed, and the cells washed three times with sterile PBS, topped up with 2–5% FBS growth media and incubated at 28, 34 or 37 °C for five days. The cell culture supernatants were harvested and stored at −80 °C, then titrated on C6/36 cells in 96-well plates, with 4 wells per ten-fold dilution per triplicate. The titration plates were incubated for 5 days at 28 °C, cells were fixed in 20% acetone, 0.02% BSA in PBS and replication was assessed by fixed-cell ELISA. Fixed cells were blocked for 30 min at RT in blocking buffer (0.05 M Tris/HCl (pH 8.0), 1 mM EDTA, 0.15 M NaCl, 0.05% (*v*/*v*) Tween-20, 0.2% *w/v* casein). Primary flavivirus NS1-reactive monoclonal antibody (mAb) 4G4 was added to each well after removing the blocking buffer and incubated at 37 °C for one hour [29]. Plates were washed with PBS containing 0.05% Tween-20 four times and secondary HRP-conjugated antibody (goat anti-mouse, Dako, Agilent Technologies, Amstelveen, The Netherlands) was added 1:2000 in blocking buffer and incubated at 37 °C for one hour. Plates were washed six times with PBS-Tween and ABTS based substrate (1 mM 2,2′-azino-bis(3-ethylbenzothiazoline-6-sulphonic acid)) with 3 mM hydrogen peroxide in a 0.1 M citrate/0.2 M Na_2_PO_4_ buffer pH 4.2) was added and left to develop in the dark at RT for one hour. Finally, the absorbance of each well was measured by an automated 96-well spectrophotometer at 405 nm. The titre obtained was determined using Reed and Muench’s guidelines [30].

## 3. Results

### 3.1. Flavivirus CpG Dinucleotide Usage

**Figure 1:**
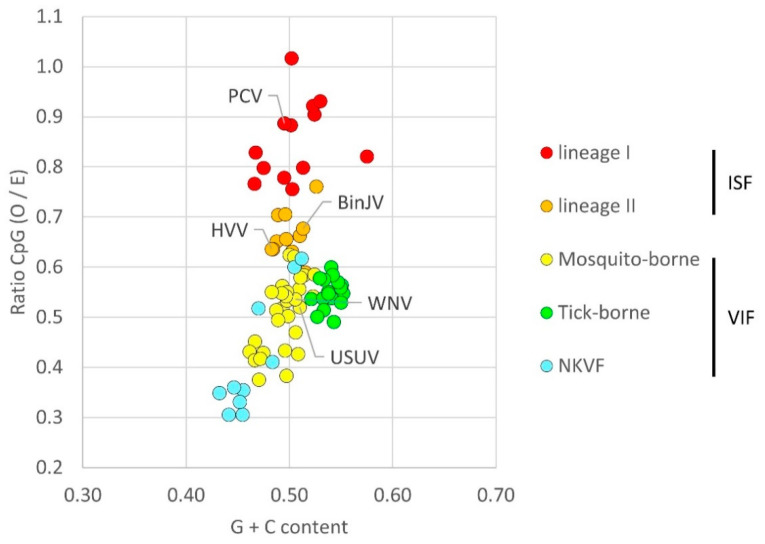
Genome composition of flaviviruses. Data points represent CpG ratio of flavivirus genomes normalised to the available mononucleotides with the expected unsuppressed frequency set to 1.0. Viruses are grouped by host range, indicated by different colours.

To determine the genome composition of recently discovered ISFs and illustrate to what extent lineage I and II ISFs suppress genomic CpG dinucleotides, the mono- and dinucleotide frequencies present in flavivirus genome sequences were analysed. The observed CpG dinucleotide frequency was normalised to the expected frequency based on mononucleotide availability (O/E). Flaviviruses were grouped as lineage I or II ISFs, and as mosquito-borne, tick-borne or NKVF VIFs (Figure 1). The genomes of all ISFs clearly display higher frequencies of CpG dinucleotides compared to VIFs. Both ISF lineages form separate clusters with limited overlap between the two groups, indicative of distinct CpG usage by both lineages, with the lineage I ISFs containing higher CpG dinucleotide frequencies (average ratio 0.86, SD 0.07 O/E) compared to lineage II ISFs (ratio 0.66, SD 0.04 O/E) (Figure 1). The open-reading frames (ORF) of an example lineage I ISF, Palm Creek virus (PCV) and the viruses used in this study were further analysed to directly compare their CpG dinucleotide usage (Table 2). Across the flavivirus ORF, there were no observable clusters of CpG dinucleotides. The lineage I ISF PCV contained most CpG dinucleotides with a total of 534, whereas lineage II ISFs BinJV and Hidden Valley virus (HVV) contained 452 and 405 CpG dinucleotides, respectively. In comparison, the VIFs Usutu virus (USUV) and close relative WNV (Kunjin) used in this study contained only 345 and 343 CpG dinucleotides, respectively (Table 2).

### 3.2. Lineage II ISF Protein Expression and RNA Replication in Human Cells Is Restricted by ZAP and Temperature

**Figure 2:**
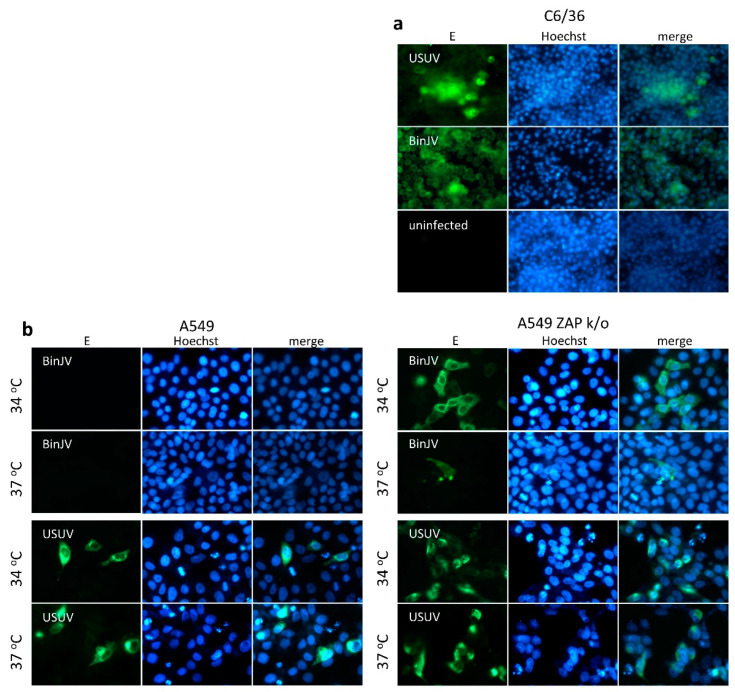
Binjari virus protein expression in human ZAP ko cells. (**a**) C6/36 mosquito cells infected with ISF BinJV and VIF USUV and incubated at 28 °C. (**b**) A549 human lung epithelial carcinoma cells and CRISPR Cas9 ZAP knockout thereof infected with BinJV or USUV and incubated at the indicated temperatures. Four days post infection, cells were fixed and viral envelope proteins were stained with cross reactive BJ-6E6 monoclonal antibodies that detect envelope (E) (green) and nuclei stained with Hoechst 33342 (blue). Micrographs were taken using a Zeiss Axio Observer Z1m inverted microscope with an LD Plan-Neofluar 40×/0.6 Ph2 korr objective and are representative images from one of three biological repeat experiments, see Figure S1 for additional images.

To investigate whether the vertebrate zinc-finger antiviral protein detects BinJV and subsequently prevents viral protein expression post entry, we made use of a ZAP knockout cell line from adenocarcinoma human alveolar basal epithelial cells (A549) origin [20]. Aedes albopictus C6/36 mosquito cell-grown stocks of virus were inoculated at a high multiplicity of infection (MOI 10) onto the wildtype (WT) and ZAP KO cell lines. Cells were incubated at either 34 or 37 °C for four days before they were fixed and stained for flavivirus envelope protein (E), using antibody BJ-6E6, which detects both VIFs and Lineage II ISFs [3,5]. The VIF USUV was used as a positive control. In mosquito cells, both BinJV and USUV readily expressed E protein throughout the cell monolayer (Figure 2a). In human A549 cells, BinJV E protein expression was only observed when ZAP was knocked out. Furthermore, at 37 °C, only incidental single cells were positive for BinJV E protein, whereas at 34 °C, clusters of cells expressing BinJV E were more readily detected (Figure 2b and Figure S1). In contrast, USUV replicated in the A549 WT and ZAP KO cells at both temperatures (Figure 2b and Figure S1).

**Figure 3:**
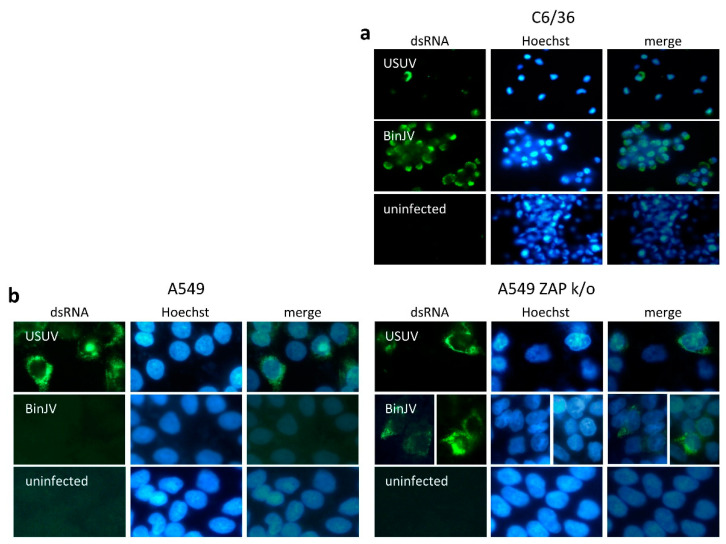
BinJV dsRNA replication intermediates in human ZAP knockout cells. (**a**) C6/36 mosquito cells and (**b**) A549 and ZAP knockout thereof infected with VIF USUV or ISF BinJV. Samples were stained for viral dsRNA (green) and nuclei stained with Hoechst 33342 (blue). Micrographs were taken using a Zeiss Axio Observer Z1m inverted microscope with an LD Plan-Neofluar 40×/0.6 Ph2 korr objective and are representative images from one of three biological repeat experiments, see Figure S2 for additional images.

Next, dsRNA replication intermediates were stained to investigate whether BinJV protein expression in human ZAP knockout cells was accompanied by viral RNA replication. C6/36 mosquito cells, A549 and A549 ZAP knockout cells were infected either with USUV or BinJV and incubated at 28 or 34 °C, respectively. Four days post infection, cells were fixed and stained for dsRNA replication intermediates. RNA replication intermediates were detected for both viruses in mosquito cells (Figure 3a and Figure S2). USUV-infected cells readily displayed cytoplasmic dsRNA both in the presence and absence of ZAP, whereas BinJV dsRNA was only detected in ZAP knockout cells (Figure 3b and Figure S2). These results confirm that BinJV can indeed enter vertebrate cells at 34 °C and identify ZAP as an antiviral host protein that subsequently limits viral protein expression and RNA replication.

### 3.3. BinJV Activity in Human Cells Is Independent of IRF-3/7 Mediated Type I IFN Responses

**Figure 4:**
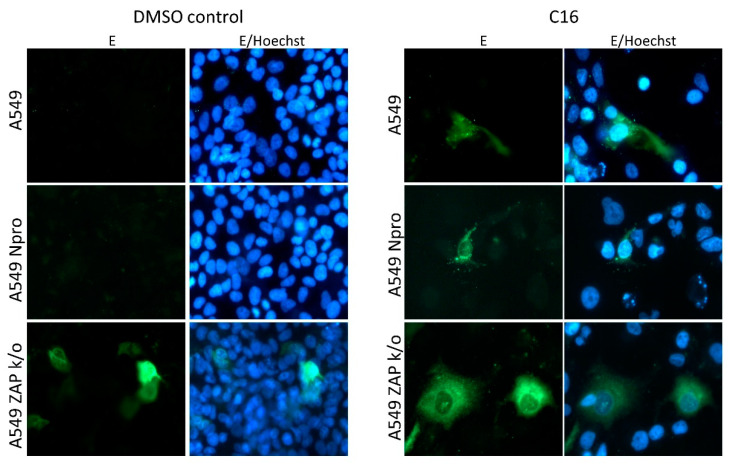
BinJV envelope expression in human cells can be established by kinase inhibitor C16 and not IRF3/7 degradation. A549 cells, ZAP k/o cells and A549 NPro expressing cells infected with BinJV and either treated with kinase inhibitor C16 or DMSO as a control. Cells were fixed and stained for BinJV envelope (E) with BJ-6E6 monoclonal antibodies (green) and nuclei stained with Hoechst 33342 (blue). Microscopic images were taken using a Zeiss Axio Observer Z1m inverted microscope with an LD Plan-Neofluar 40×/0.6 Ph2 korr objective.

In the absence of IFN stimulation base levels of ZAP are constitutively expressed and detectable in A549 cells [31]. However, ZAP expression is increased upon induction with type I IFNs, which is particularly evident after IFN regulatory factor 3 (IRF-3) signalling [31,32]. To investigate whether the IRF-3/7 mediated IFN response limits BinJV in vertebrate cells, A549 cells modified to express bovine viral diarrhoea virus (BVDV, genus *Pestivirus*) N-terminal protease fragment (NPro), were used, in addition to the wildtype A549 and ZAP knockout cells. The expression of BVDV NPro inhibits the activity of IRF-3 and IRF-7 [33,34]. Cells were infected with BinJV at 34 °C and stained for E protein expression. BinJV E protein was only detected in the ZAP knockout cells (Figure 4, left panel), suggesting that inhibition of IRF-3/7 signalling does not sufficiently reduce ZAP expression and other relevant antiviral responses to allow protein expression from the BinJV RNA. The antiviral activity of ZAP is additionally dependent on phosphorylation by cellular glycogen synthase kinase 3β (GSK3β) [35]. Kinase inhibitor C16 inhibits the phosphorylation of ZAP and rescues replication of CpG-high viruses that are otherwise attenuated [20,24,36]. Indeed, treatment with C16 resulted in low levels of BinJV E protein expression in both parental A549 and A549 NPro expressing cell lines (Figure 4, right panel) that was not observed in mock (DMSO) treated samples (Figure 4, left panel). Together, these results indicate that a functional ZAP-mediated antiviral response is the primary barrier that inhibits BinJV replication in human cells independent of IRF3/7-mediated type I IFN responses.

### 3.4. BinJV Produces Infectious Progeny in Human ZAP Knock out Cells

**Figure 5:**
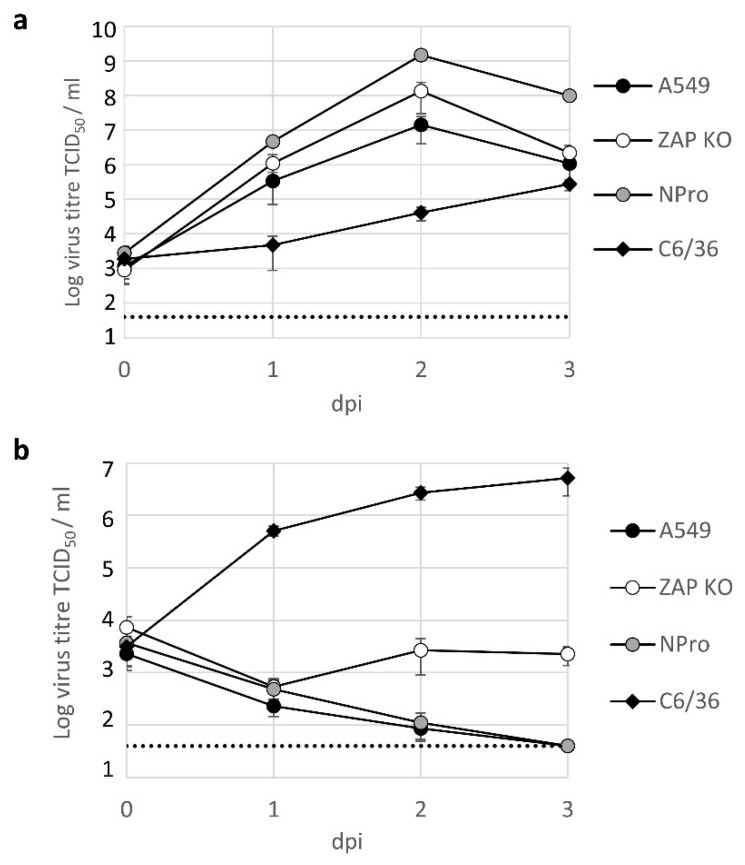
BinJV replication in ZAP k/o cells. Growth curves of USUV (**a**) and BinJV (**b**) in C6/36 mosquito cells (black diamonds), human A549 cells (black circles), A549 ZAP k/o cells (open circles) and A549 NPro cells (grey circles). Samples were taken on the indicated days post infection (dpi). Data points represent the average of independent biological experiments and error bars represent one standard error of the mean. Dotted line indicates the detection limit.

To investigate whether BinJV protein translation and RNA replication in the absence of ZAP can lead to the assembly of new virus particles that successfully bud from the infected cell, we quantified progeny virus from cell culture supernatant. Cell lines were infected with USUV or BinJV at 28 (C6/36) or 34 °C (A549) at MOI 1. After two hours, the inoculum was removed, cells were washed, and cell culture supernatant was sampled at 24-h intervals. All samples were titrated on permissive C6/36 mosquito cells. As expected, USUV readily produced progeny virus in all cell types tested, albeit at a slower rate in mosquito cells compared to human cells, which is expected for this virus (Figure 5a) [37]. BinJV replicated to high titres in mosquito cells, while infectious virus titres rapidly declined in the cell culture supernatant of wildtype A549 cells or A549 NPro cells, indicative of a lack of replication in these cells. However, infectious virus titres increased in the supernatant from ZAP KO cells at two days post infection compared to the previous day. BinJV titres reached an average tissue culture infectious dose 50% (TCID_50_) of 2.7 × 10^3^ TCID_50_/mL (SEM ± 1.8 × 10^3^) and 2.2 × 10^3^ TCID_50_/mL (SEM ± 8.9 × 10^2^) on day 2 and 3 post infection, respectively (Figure 5b).

**Figure 6:**
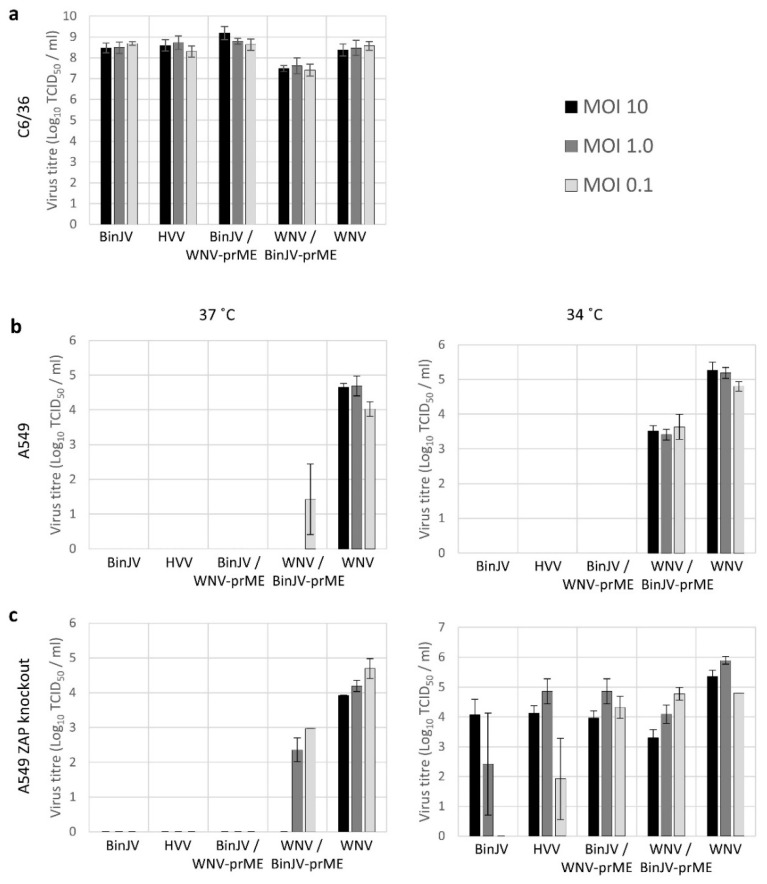
Lineage II ISF replication in human cells is regulated by ZAP and temperature at multiple stages of infection. C6/36 mosquito cells (**a**), human A549 cells (**b**), or A549 ZAP knockout cells (**c**) were infected with three different multiplicity of infections (MOI). After five days of incubation at the indicated temperatures infectious virus titres in the cell culture supernatant was measured. Bars represent average values of triplicate samples from the indicated MOI infection and error bars one standard deviation of the mean.

Next, we investigated whether other lineage II ISFs are similarly able to infect vertebrate host cells. As a second lineage II ISF we employed the recently discovered HVV. To examine whether the effects of vertebrate physiological temperatures act on ISF structural proteins alone or also affect non-structural protein function and to further disentangle the temperature sensitive phenotype from restriction by vertebrate ZAP we used previously constructed chimeric viruses [5]. The chimeric viruses either contained the structural genes (prM-E) from BinJV in a VIF WNV Kunjin backbone or vice versa; WNV prM-E in a BinJV backbone (see Table 2 for CpG usage). Cells were infected with three different MOIs and the presence of progeny virus in the cell culture supernatant was assessed five days post inoculation. All viruses grew to high titres on mosquito cells (>10^8^ TCID_50_/mL), except WNV/BinJV-prME which produced approximately 10-fold lower titres of progeny virus compared to both WT BinJV and WNV (Figure 6a). At 37 °C, WNV titres reached 10^4^–10^5^ TCID_50_/mL in both WT and ZAP KO A549 cells. At this temperature, only minute quantities of the WNV/BinJV-prME chimera were detected during some of the experimental repeats in the supernatant of human A549 cells (Figure 6b, left). In contrast, infectious progeny virus from WNV/BinJV-prME was consistently detected in the wildtype A549 cells at 34 °C indicating that particles with BinJV prM-E more effectively infect human cells at temperatures below 37 °C (Figure 6b, right). Slightly higher WNV/BinJV-prME progeny virus titres were detected in A549 ZAP KO cells at 37 °C compared to wildtype A549 cells (Figure 6c, left). Importantly, progeny virus was detected at 34 °C for all viruses in A549 ZAP knockout cells (Figure 6c, right). Inoculation with high MOI improved infection of A549 ZAP knockout cells with BinJV and HVV, while both chimeras produced relatively high titres after infection at low MOI (0.1) (Figure 6c). Together, these results indicate that lineage II ISF replication in vertebrate cells is inhibited by ZAP and that both virus entry and subsequent RNA replication are strongly temperature dependent.

## 4. Discussion

Vertebrate cells are protected from infection by insect-specific flaviviruses by barriers and incompatibilities that block ISF replication at multiple stages of the cellular infection cycle. This is particularly apparent for lineage I ISFs [10,11]. However, the close phylogenetic relationship between lineage II ISFs and VIFs suggests that lineage II ISFs are better suited to infect vertebrate cells than the more distant lineage I ISFs. Indeed, BinJV structural genes prM-E allow entry into vertebrate cells [3]. The lack of productive lineage II ISF replication in vertebrate cells suggests that additional barriers inhibit subsequent steps in the infection cycle, potentially by the activation of antiviral responses [3]. Here, we showed for the first time that lineage II ISFs can successfully replicate in human ZAP knockout cells. Although the infectious BinJV titres measured in the supernatant of human A549 ZAP knockout cell culture remained below that of the initial inoculum, an increase in viral titres was detected at two and three days post infection compared to one day post infection. In contrast, in the parental ZAP expressing cells or the IFN incompetent NPro expressing cells residual virus from the inoculum declined until it was no longer detected (Figure 5b). Similarly, at five days post infection 10^3^ to 10^5^ TCID_50_/mL of BinJV, HVV and BinJV/WNV-prME was only measured in the supernatant from ZAP knockout cells at 34 °C and not in the ZAP expressing cell line (Figure 6). In contrast, WNV infection of ZAP knockout cells did not result in higher virus titres compared to infection of ZAP expressing cells, suggesting that ZAP predominantly affects the replication of viruses with an ISF backbone (Figure 6). Together, these results show that lineage II ISFs replication in vertebrate cells is inhibited by physiological temperatures and ZAP.

### 4.1. ZAP Senses Non-Self RNA

Vertebrate ZAP is involved in the detection of CpG dinucleotides in (viral) RNA [19,20,21,22]. ZAP can directly influence translation initiation of RNA through the sequestration of eukaryotic translation initiation factor 4A (eIF4A) [38], or via its interaction with stress granule and processing body components and the 3′-5′ exosomal and the 5′-3′ XRN1-mediated RNA degradation pathways [39,40,41,42]. Artificially increasing CpG dinucleotides in vertebrate viruses consistently results in attenuated virus replication [19,24,26]. Attenuation of Echovirus 7 (E7) (family *Picornaviridae*, genus *Enterovirus*) was shown to occur immediately post viral entry. The degree of attenuation correlated to the total number of CpG dinucleotides that were introduced to the point where virus replication was no longer observed [36]. Similarly, the chimeric virus with BinJV-prME and a WNV backbone was able to replicate in wildtype A549 cells, specifically at 34 °C. However, replication of this chimeric virus was significantly improved in ZAP knockout cells (Figure 6), suggesting that the additional 21 CpG dinucleotides in the prM-E RNA sequence of BinJV compared to WNV (Table 2) may result in detection by ZAP and attenuated replication in wildtype A549 cells. This supports our initial hypothesis that the relatively high CpG dinucleotide frequencies in the genomes of ISFs restrict virus replication in vertebrate cells. However, we acknowledge that the experiments presented here do not provide direct evidence of a causal relationship between the observed ZAP-mediated restriction of BinJV and HVV and their genomic CpG dinucleotide frequencies.

### 4.2. Antiviral Responses

The antiviral activity of ZAP is enhanced by the IFN response [31,43] and is additionally associated with a number of recently identified host cell proteins, including RNase L [20,44,45]. Here, we report that the ZAP-mediated immune response is an important barrier that inhibits BinJV replication in human cells independent of IRF3/7-mediated type I IFN responses (Figure 4 and Figure 5). In a previous study, infections in MEF cells with either IFNAR or RNase L knocked out displayed some BinJV protein expression [3]. This suggests that the antiviral activity of ZAP against ISFs may be enhanced by IFN induced responses such as the RNase L pathway similar to what was observed in previous experiments with E7 [20]. In addition to the absence of ZAP, the observed influence of temperature on the requirements for replication of BinJV, HVV and the two chimeras suggests that both attachment and/or entry of particles with BinJV prM-E and subsequent virus replication are more efficient at temperatures below that of most warm-blooded vertebrates (<37 °C) (Figure 2 and Figure 6). Additionally, the functionality of cellular antiviral proteins other than ZAP is reduced at lower temperatures [46], which may contribute to allow ISF replication in the absence of ZAP. It is interesting to note that in the case of WNV/BinJV-prME, titres of virus progeny decreased as the MOI increased (Figure 6). While the reasons for this trend are not yet clear, one hypothesis could be that this chimeric virus triggers stronger antiviral responses at higher MOI.

### 4.3. Lineage II ISFs

Co-evolution of virus and host fuels the arms-race between host antiviral responses and viral counter defense strategies [47]. VIFs alternately infect evolutionary distant vertebrate hosts and invertebrate vectors, which suggests that VIFs encounter distinct evolutionary pressures that together constrain their diversity [27,48]. The low vertebrate-like CpG dinucleotide frequencies of VIFs (Figure 1) and the strong antiviral activity of ZAP against BinJV and HVV (Figure 2, Figure 3, Figure 4, Figure 5 and Figure 6) suggests reduced CpG frequencies are required for flaviviruses to successfully infect vertebrate cells. In a recent study we experimentally demonstrated opposing selection pressures on CpG dinucleotide usage in flavivirus genomes exist between the vertebrate host and invertebrate vector. CpG-high synonymous mutants of the VIF Zika virus were attenuated in vertebrate cells and mice, whereas the same mutant viruses replicated to higher titres in mosquito cells and displayed more effective dissemination in live mosquito vectors compared to wildtype Zika virus [49]. This may also explain why BinJV/WNV-prME chimeras grow to higher titres in mosquito cells in comparison to WNV [5]. Thus, repeated replication in the invertebrate may drive the accumulation of CpG dinucleotides, which gives rise to a genome more suitable for an insect-specific host-range. A relatively recent evolutionary split from a common VIF ancestor has been suggested [1] and can explain both the close phylogenetic relationship between VIFs and lineage II ISFs and the relatively mild suppression of CpG dinucleotides that is still observed in lineage II ISFs (Figure 1).

The diverse and largely unknown virome present in invertebrates and their sometimes close relationship with vertebrate-infecting viruses inevitably raises the question whether insect-specific viruses, here specifically ISFs, can make the jump into mammals and evolve to become VIFs [50]. For the genus *Flavivirus* this seems highly unlikely as the complete absence of ISF replication in vertebrate cells would require multiple adaptations to resolve. Adaptations that enable infections at higher temperatures (i.e., 37 °C) have been shown to occur relatively quickly for some flaviviruses under experimental conditions [51,52]. Serial passaging of ISFs in human ZAP knockout cells could identify some of the additional genetic changes required for ISFs to more efficiently replicate in human cells. However, even lineage II ISFs would additionally need to evolve to either avoid or inhibit ZAP-mediated immunity. In recent years there has been an increasing interest in ISFs for their close relationship to pathogenic VIFs and potential to interfere with VIFs [53] or to be used as vaccine platforms [5]. The observation that ZAP is a strong barrier for lineage II ISF replication in vertebrate cells provides additional rationale for the safe use of chimeric viruses with an ISF backbone to express VIF structural proteins as part of a vaccine platform.

## Figures and Tables

**Table 1:** Accession numbers of flavivirus ORFs used in this study.

	Mosquito-Borne VIFs	NKVF VIFs	Lineage I ISFs	Lineage II ISFs
DQ235145	KF917535	NC_008718	NC_005064	NC_024017
AY323490	AY632536	NC_026624	JQ268258	MF139576
AF331718	AY632538	NC_005039	NC_024299	NC_017086
AF253419	KF917538	AF160193	NC_027817	NC_016997
NC_001809	DVU88536	NC_026620	NC_001564	KC692068
DQ235152	U88536	AJ242984	GQ165809	KC496020
DQ235151	M19197	AJ299445	NC_012932	MF139575
DQ235153	M93130	KJ469370	KC505248 (PCV)	NC_024805
AY323489	AF326573	NC_034007	NC_012671	EU159426
L06436	LN849009	AF144692	HE574574	MG587038 (BinJV)
DQ235149	M18370		NC_008604	MN954647 (HVV)
TEU27495	EU082200		KX669689	
KU761576	AY898809		DQ400858	
L40361	AF161266		NC_033694	
DQ235144	DQ525916			
DQ235150	AY453411 (USUV)			
DQ235148	D00246 (WNV_KUN_)			
DQ235146	M12294			
	AF013413			
	NC_009029			
	MF380434			
	NC_009028			
	MF461639			
	KC734552			
	NC_018705			
	KF192951			
	DQ859059			
	DQ859063			
	DQ859058			
	X03700			

**Table 2:** CpG dinucleotide frequencies of viruses used in this study.

Viruses	Group ^a^	Total Bases		G + C Content (%)		CpG ^b^		CpG O/E ^c^	
		ORF	prM-E	ORF	prM-E	ORF	prM-E	ORF	prM-E
PCV	Lineage I ISF	10,092	ND	50	ND	534	ND	0.89	ND
BinJV	Lineage II ISF	10,299	2004	51	50	452	82	0.68	0.66
HVV	Lineage II ISF	10,299	ND	50	ND	405	ND	0.63	ND
USUV	VIF	10,302	ND	51	ND	345	ND	0.51	ND
WNV	VIF	10,299	2004	51	51	343	61	0.53	0.48
WNV/BinJV-prME	N/A	10,299	2004	51	50	364	82	0.56	0.66
BinJV/WNV-prME	N/A	10,299	2004	51	51	431	61	0.64	0.48

^a^ Groups based on phylogenetic diversity and host range, with insect-specific flaviruses (ISF) lineage 1 or 2 and mosquito-borne vertebrate infecting flaviviruses (VIF). ^b^ Total CpG dinucleotides in the ORF or prM-E. ^c^ CpG O/E refers to the ratio of observed/expected CpG frequency. Expected CpG frequency is based on the mononucleotide availability. N/A: not applicable. ND: not done.

## Supplementary Materials

The following are available online at https://www.mdpi.com/article/10.3390/v13040573/s1, Figure S1: Binjari virus protein expression in human ZAP knockout cells, Figure S2: Binjari virus dsRNA replication intermediates in human ZAP knockout cells.
