# Supplementary material for: Insect-Specific Flavivirus Replication in Mammalian Cells Is Inhibited by Physiological Temperature and the Zinc-Finger Antiviral Protein
Source: Viruses. 2021 Mar 29;13(4):573. doi: 10.3390/v13040573 (PMC8066048; doi:10.3390/v13040573)
Supplement: Supplementary file 1 [file viruses-13-00573-s001.pdf]

viruses

MDPI

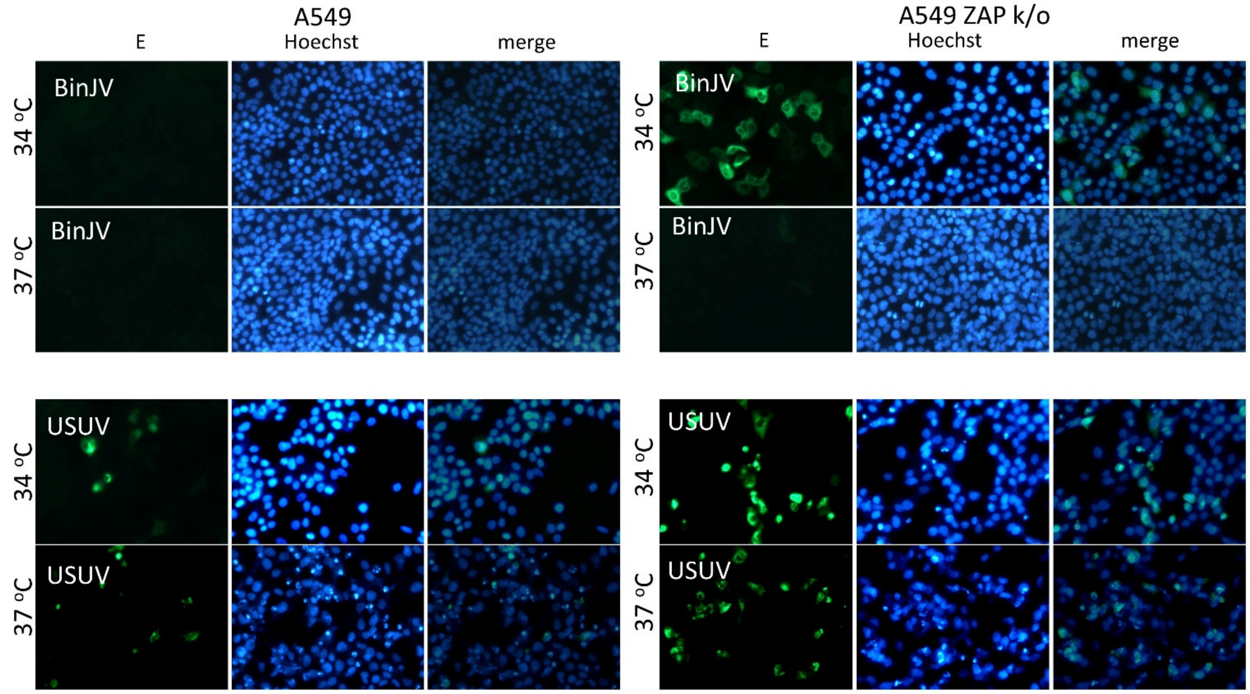

Figure S1. Binjari virus protein expression in human ZAP knockout cells.

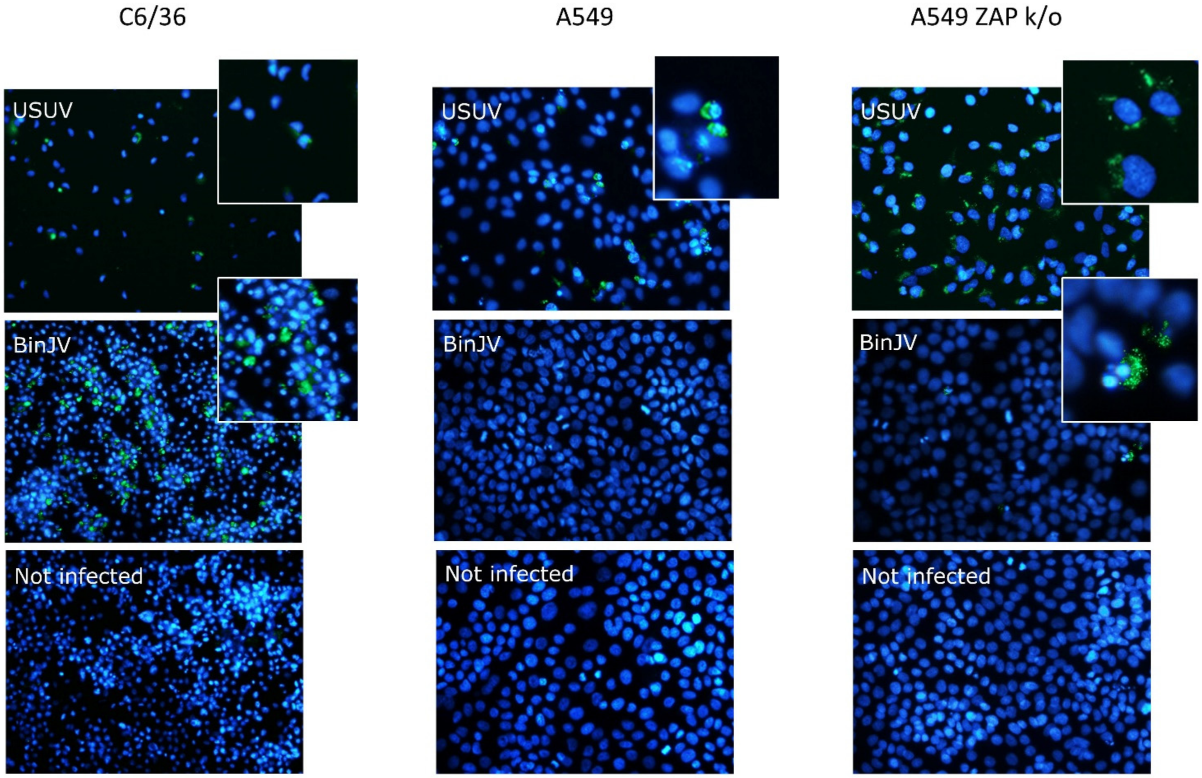

Figure S2. Binjari virus dsRNA replication intermediates in human ZAP knockout cells.
